# Bacterial Quorum Sensing Molecules Promote Allergic Airway Inflammation by Activating the Retinoic Acid Response

**DOI:** 10.1016/j.isci.2020.101288

**Published:** 2020-06-20

**Authors:** Renlan Wu, Xingjie Li, Ning Ma, Xiufeng Jin, Xiefang Yuan, Chen Qu, Hongmei Tang, Zhigang Liu, Zongde Zhang

**Affiliations:** 1 Inflammation & Allergic Diseases Research Unit, Affiliated Hospital of Southwest Medical University, Luzhou, Sichuan 646000, China; 2 State Key Laboratory of Respiratory Disease for Allergy at Shenzhen University, Shenzhen University School of Medicine, Shenzhen 518060, China; 3 The School of Basic Medical Sciences, Southwest Medical University, Luzhou, Sichuan 646000, China; 4 Model Animal Research Center, Nanjing University, Nanjing 210061, China

**Keywords:** Biological Sciences, Immunology, Immune Response

## Abstract

IgE and IgG1 production in the type 2 immune response is the characteristic feature of an allergic reaction. However, whether bacterial molecules modulate IgE and IgG1 production remains obscure. Here, we demonstrate that the bacterial quorum sensing molecules acyl homoserine lactones (AHLs) induce IgE and IgG1 production by activating the RARE (retinoic acid response element) response in dendritic cells (DCs) *in vivo*. DC-specific knockout of the retinoic acid transcriptional factor Rara diminished the AHL-stimulated type 2 immune response *in vitro*. AHLs altered DC phenotype, upregulated OX40L and IFN-I signature, and promoted T helper 2 cell differentiation *in vitro*. Finally, AHLs activated the RARE response by inhibiting AKT phosphorylation *in vitro*, as the AKT agonists IGF-1 and PDGF abolished the effect of AHLs on the RARE response. This study demonstrates a mechanism by which AHLs drive allergic airway inflammation through activating retinoic acid signaling in DCs.

## Highlights

• Acyl homoserine lactones (AHLs) exacerbate allergic airway inflammation
• AHLs induce IgE and IgG1 production
• AHLs activate the RARE response in dendritic cells
• AHLs inhibit AKT phosphorylation

## Graphical Abstract

**Figure:**
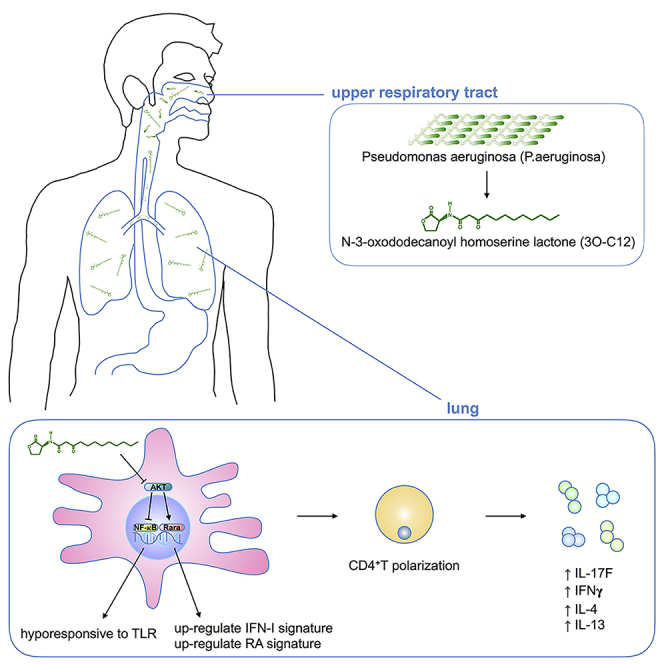

## Introduction

The prevalence of allergic diseases has increased significantly over the past decades. It has been suggested that “allergic diseases could be prevented by infection, exposure, or colonization of microorganisms in the neonatal period” (Bisgaard et al., 2011; Fyhrquist et al., 2014; Lambrecht and Hammad, 2017; Smits et al., 2016; Strachan, 1989). However, it has also been demonstrated that specific bacterial infection or colonization is a risk factor for food allergies and asthma (Huang and Boushey, 2015; Savage et al., 2018). This paradox reveals the differential role of microbes in shaping the allergic response of the host. There is evidence that exposure to farm dust enriched with bacterial endotoxin can reduce the severity of asthma (Schuijs et al., 2015). Furthermore, gut flora metabolite short-chain fatty acids decrease the circulating level of immunoglobulin E (IgE) and thus ameliorate allergic lung inflammation (Cait et al., 2018). However, whether bacteria-derived molecules can promote allergic reactions remains elusive.

The bacterium *Pseudomonas aeruginosa* causes a wide range of opportunistic infections among immunocompromised patients and is also a commensal bacteria that colonizes the human gut and upper respiratory tract (Huang et al., 2011; Okuda et al., 2010; Stoodley and Thom, 1970). *P. aeruginosa* has been found in patients with asthma (Huang et al., 2011; Li et al., 2017), and its colonization in a patient with chronic rhinosinusitis was correlated with an oncoming attack of asthma (Cleland et al., 2013; Zhang et al., 2015). Furthermore, persistent colonization of *P. aeruginosa* is associated with a positive skin prick test reaction to multiple allergens (Clarke et al., 1981; Pitcher-Wilmott et al., 1982). The mechanisms by which *P. aeruginosa* provokes allergic reactions have not been determined yet.

One specific characteristic of *P. aeruginosa* is to use diffusible acyl homoserine lactones (AHLs) as quorum sensing molecules for inter- and intra-species communication (Khajanchi et al., 2011; Miyairi et al., 2006; Passador et al., 1993; Tang et al., 1996). One of the AHLs, N-3-oxododecanoyl homoserine lactone (3O-C12), has been demonstrated to possess immunomodulatory properties such as inhibition of lymphocyte proliferation and downregulation of T helper 1 (Th1) cytokine interleukin (IL)-12 production (Telford et al., 1998). This suggests that 3O-C12 may promote Th2 differentiation in the allergic response.

Dendritic cells (DCs) are the most effective antigen-presenting cells. After engulfing an antigen, DCs stimulate CD4+ T cells toward Th1 or Th2 differentiation, which leads to specific antibody production in B cells. Previously, we demonstrated that commensal fungi in the gut are essential for maintaining DC retinoic acid (RA) signaling in lymphoid tissue (Zhang et al., 2016). However, overgrowth of commensal fungi in the gut induced M2 macrophage polarization and exacerbated pulmonary allergic reactions (Kim et al., 2014; Skalski et al., 2018). Furthermore, it has been demonstrated that RA can promote M2 macrophage polarization (Chen et al., 2019; Vellozo et al., 2017) and boost Th2 cytokine production (Dawson et al., 2008; Lovett-Racke and Racke, 2002), which are critical for class switching recombination of IgE and IgG1. These studies imply a link between microorganisms, RAs, and allergic reactions. Furthermore, another study has indicated that intestinal microbiota can modulate RA signaling in intestinal epithelial cells (Bhattacharya et al., 2016). These findings led us to investigate whether bacteria-derived 3O-C12 could modulate RA signaling in DCs, which may contribute to the type 2 immune response.

Allergen-specific IgE and IgG1 production is the hallmark of allergic diseases. T helper 2 (Th2) cytokines such as IL-4 and IL-13 are required for class switching recombination of IgE and IgG1 (Gould and Sutton, 2008). After engulfing antigens, DCs can stimulate CD4+ T cells toward Th2 differentiation by multiple mechanisms involving the DC surface protein OX40L (Flynn et al., 1998; Ito et al., 2005; Kaisar et al., 2018; Ohshima et al., 1998); transcription factors IRF4, IL-10, and IL-33 (Gao et al., 2013; Williams et al., 2013); and the recently identified DC-intrinsic type I interferon signature (Connor et al., 2017; Janss et al., 2016; Webb et al., 2017). However, whether bacteria-derived molecules engage these DC genes and prime CD4+ T cell Th2 differentiation remains incompletely understood.

In this study, we demonstrate that the bacterial quorum sensing molecule 3O-C12 stimulates IgE and IgG1 production by provoking the DC RARE (retinoic acid response element) response. 3O-C12 inhibits Toll-like receptor (TLR)-induced DC maturation but activates type I interferon and OX40L by the RA signal transcription factor retinoic acid receptor α (Rara). This study sheds light on the important roles of bacterial diffusible AHL molecules in promoting host allergic reactions via DC RA signaling.

## Results

### 3O-C12 Stimulates Allergic Lung Inflammation

**Figure 1:**
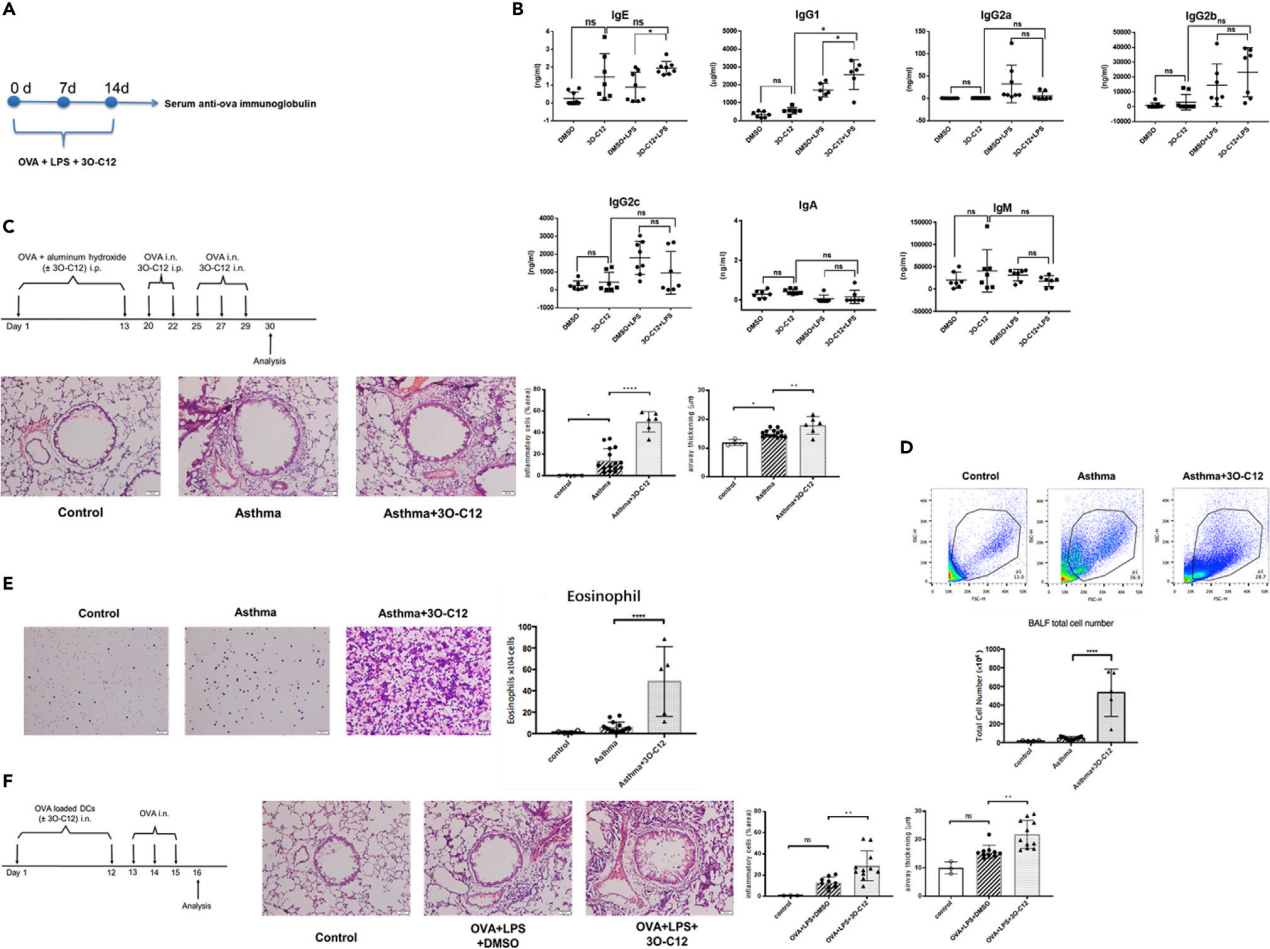
3O-C12 Stimulates OVA-Specific IgE and IgG1 Production and Enhances Allergic Lung Inflammation (A) Graphical representation of mouse immunization. (B) Wild-type C57BL/6 mice (6–8 weeks, male and female) were subcutaneously immunized with OVA (50 μg/mouse) + LPS (0.5 μg/mouse) with or without 3O-C12 (250 μg/mouse) every week for 3 weeks; incomplete Freund adjuvant was used as a vehicle. Mouse serum was collected; OVA-specific IgE, IgG1, IgG2a, IgG2c, IgM, IgA, and IgG2b were analyzed with ELISA. Results are representative of three independent experiments. (C–E) (C) BALB/c mice were divided into three groups: control (n = 4), asthma (n = 15), and asthma + 3O-C12 (n = 6). The mice were intraperitoneally (i.p.) injected on days 1 and 13 with OVA (50 μg) and aluminum hydroxide (2 mg). On days 20 and 22, the mice were intranasally (i.n.) challenged with OVA (40 μg) and i.p. administered with or without 3O-C12 (500 μg). On days 25, 27, and 29, the mice were i.n. challenged with OVA (40 μg) and i.n. administered with or without 3O-C12 (50 μg). One day after the last OVA challenge, mice were sacrificed and assessed for lung histology (scale bar, 50 μm) (C) and bronchoalveolar lavage fluid (BALF) total cell number (D) and eosinophil number (E). (F) BMDCs from C57BL/6 mice were stimulated by OVA (200 μg/mL) and LPS (100 ng/mL) overnight, with or without 3O-C12 (10 μM). BMDCs were transferred i.n. to recipients (C57BL/6 mice) (n = 3 in control group; n = 10 in OVA + LPS + DMSO group; n = 11 in OVA + LPS + 3O-C12 group) on days 1 and 12. Each recipient was challenged with 40 μg OVA i.n. on days 13, 14, and 15. One day after the last OVA challenge, mice were sacrificed and assessed for lung histology (scale bar, 50 μm). The statistical quantification (inflammatory cells and airway thickening) of Figures 1C and 1F was analyzed by ImageJ software. Data are presented as mean ± SD; p values were calculated using the two-tailed Student's t test. Data that did not exhibit a normal distribution were analyzed using the nonparametric Kruskal-Wallis test with Dunn's post hoc test. ∗p < 0.05, ∗∗p < 0.01, ∗∗∗p < 0.001, ∗∗∗∗p < 0.0001; n.s., not significant.

The production of specific IgE and IgG1 in the type 2 immune response is characteristic of allergic diseases. Colonization of *P. aeruginosa* is highly correlated with the development of an allergic reaction (Clarke et al., 1981; Cleland et al., 2013; Zhang et al., 2015). To test the possibility that *P. aeruginosa-*derived 3O-C12 promotes allergen-specific antibody production, wild-type C57BL/6 mice were immunized with OVA and 3O-C12 for 3 weeks (Figure 1A). 3O-C12 was derived from the gram-negative bacteria *P. aeruginosa.* The outer membrane of gram-negative bacteria is largely made of lipopolysaccharide (LPS), which suggests that 3O-C12 may activate an immune response together with LPS. Indeed, LPS has been used as an adjuvant in studying DC priming of the Th1-Th2 response (Gao et al., 2013). To mimic the genuine biological scenario, we also added LPS to OVA and 3O-C12 for mice immunization. 3O-C12 alone or with LPS significantly increased serum OVA-specific IgE and IgG1, slightly increased OVA-specific IgA and IgG2b, and moderately decreased OVA-specific IgG2a, IgG2c, and IgM production (Figure 1B). 3O-C12 alone or with LPS changed the DC phenotype and promoted IL-4, IL-5, and IL-13 production in CD4^+^ T cells (Figures S1E and S1F). Next, we examined whether 3O-C12 administration can increase allergic lung inflammation in a mouse model of asthma. 3O-C12 increased the total number of infiltrated cells (Figure 1D), including eosinophils (Figure 1E) in the bronchoalveolar lavage fluid; the cytokine response of CD4^+^T cells (Figure S1B); and airway inflammation (Figure 1C). 3O-C12 changed the surface marker expression of spleen-derived DCs (Figure S1A). Intranasal transfer of OVA-loaded, 3O-C12-activated bone marrow-derived dendritic cells (BMDCs) induced OVA-specific IgE (Figure S1C), higher levels of cytokines by splenic CD4^+^T cells (Figure S1D), and airway inflammation (Figure 1F). These data reveal the stimulative role of 3O-C12 on allergic lung inflammation.

### 3O-C12-Activated DCs Are Hyporesponsive to TLR Stimulation and Induce the RARE Response

**Figure 2:**
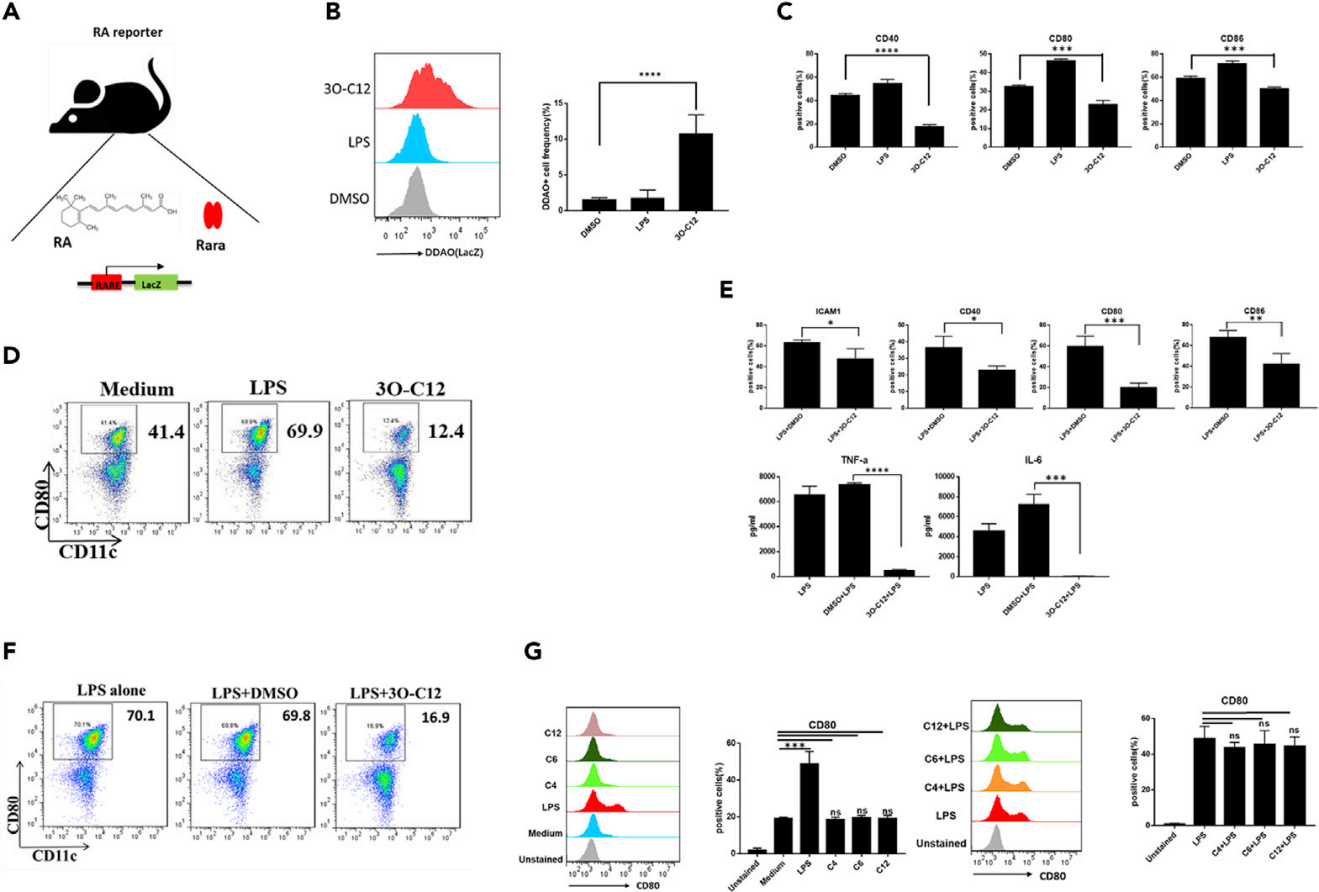
3O-C12 Activates the RARE Response (A) Graphical representation of the working model of RA reporter mice. (B) BMDCs (5–7 days) cultured from RA reporter mice were stimulated with 3O-C12 (10 μM) or LPS (100 ng/mL) for 1–2 h and incubated with DDAOG (10 μM). DDAO fluorescence was analyzed by flow cytometry. Data are representative of three independent experiments (n = 5–7). (C) BMDCs (5–7 days) cultured from RA reporter mice were stimulated with 3O-C12 (10 μM) or LPS (100 ng/mL) overnight. Expression levels of CD40, CD80, and CD86 were determined by flow cytometry; cells were gated on CD11c-positive cells. Data are representative of three independent experiments (n = 5–7). (D) DC Magnetic-activated cell sorting (MACS) isolated from the spleen of C57BL/6 mice were stimulated with LPS (100 ng) or 3O-C12 (10 μM) or were unstimulated (medium). CD80 expression was determined by flow cytometry. Data are representative of three independent experiments (n = 5–7). (E) BMDCs (5–7 days) cultured from RA reporter mice were stimulated with LPS (100 ng/mL) overnight, with or without 3O-C12 (10 μM). Expression levels of ICAM1, CD40, CD80, and CD86 were determined by flow cytometry. Cytokine levels of IL-6 and TNF-α were evaluated by ELISA. Data are representative of three independent experiments (n = 5–7). (F) DC MACS isolated from the spleen of C57BL/6 mice were stimulated with LPS (100 ng) with or without 3O-C12 (10 μM) overnight. CD80 expression was determined by flow cytometry. Data are representative of three independent experiments (n = 5–7). (G) BMDCs (5–7 days) cultured from RA reporter mice were stimulated with C4 (10 μM), C6 (10 μM), and C12 (10 μM) alone or plus LPS. CD80 expression was determined by flow cytometry. Data are representative of three independent experiments (n = 5–7). Data are presented as mean ± SD; p values were calculated using two-tailed Student's t test. ∗p < 0.05, ∗∗p < 0.01, ∗∗∗p < 0.001, ∗∗∗∗p < 0.0001; n.s., not significant.

**Figure 3:**
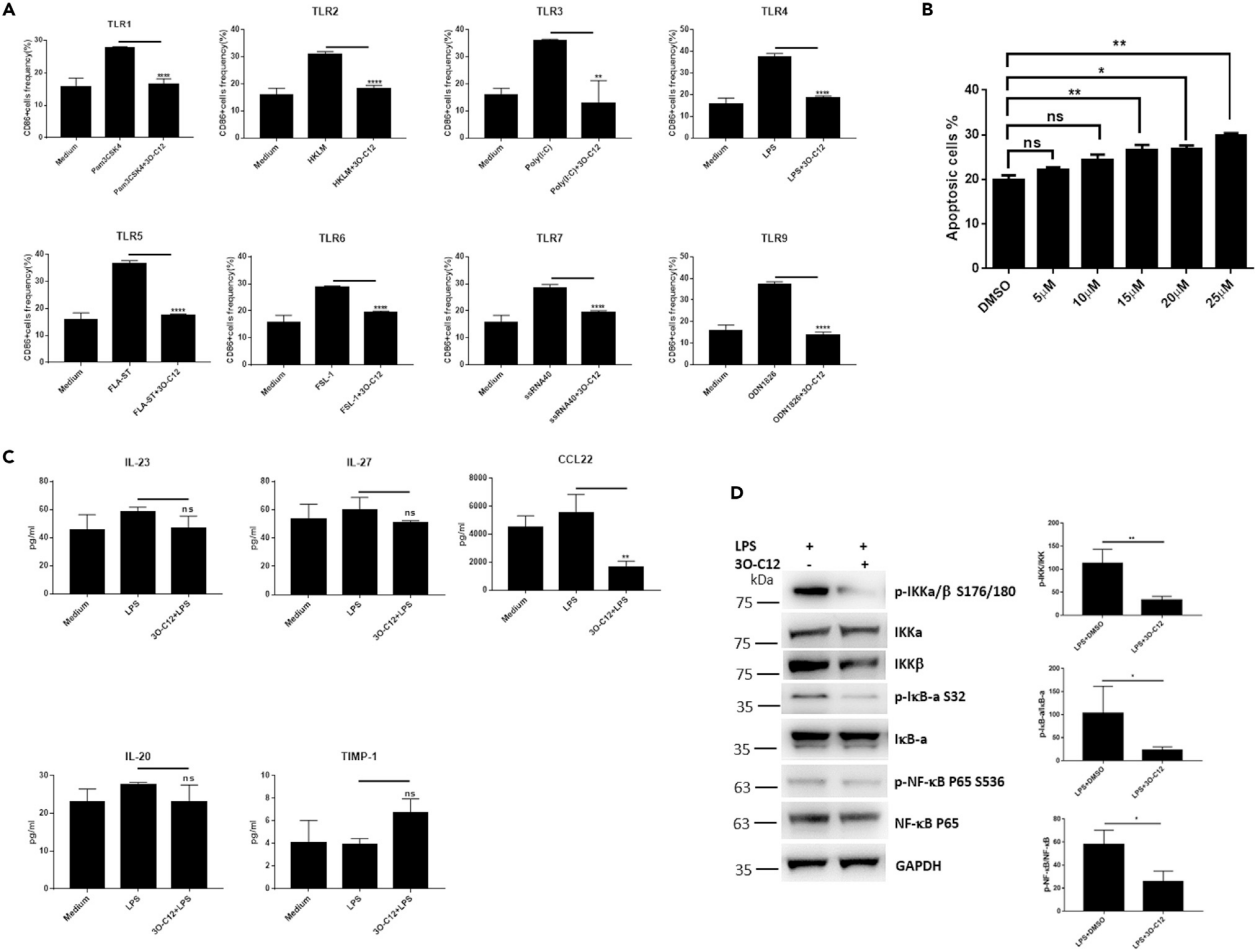
3O-C12 Inhibits TLR-Induced DC Activation (A) BMDCs (5–7 days) cultured from C57BL/6 mice were stimulated with different TLR ligands with or without 3O-C12 (10 μM) overnight. CD86-positive cells were estimated by flow cytometry. Data are representative of three independent experiments (n = 5–7). (B) BMDCs (5–7 days) cultured from C57BL/6 mice were incubated with different doses of 3O-C12 for 12 h; annexin V-positive cells were determined by flow cytometry. Data are representative of three independent experiments (n = 5–7). (C) BMDCs (5–7 days) cultured from C57BL/6 mice were unstimulated (medium) or stimulated with LPS or LPS + 3O-C12 (5 μM) overnight. Cytokines were determined by MILLIPLEX Multiplex Assays. Data are representative of three independent experiments (n = 5–7). (D) BMDCs (5–7 days) cultured from C57BL/6 mice were incubated with LPS plus 3O-C12 (10 μM) for 3 h. Cells were collected and lysed and activation of the NF-κB pathway was determined by western blot (n = 4). Data are presented as mean ± SD; p values were calculated using the two-tailed Student's t test. ∗p < 0.05, ∗∗p < 0.01, ∗∗∗∗p < 0.0001; n.s., not significant.

Next, we investigated whether bacteria-derived 3O-C12 could modulate RA signaling in DCs, which may contribute to the type 2 immune response. We used RA reporter transgenic mice that bear a β-galactosidase (LacZ) reporter under the control of a RARE (Figure 2A), and thus β-galactosidase activity could be measured by DDAO galactoside (DDAOG) to DDAO conversion. We stimulated BMDCs and spleen-derived DCs (spDCs) isolated from the transgenic mice with 3O-C12. BMDCs exposed to 3O-C12 exhibited increased DDAO fluorescence (Figure 2B). However, the DC activation markers CD40, CD80, and CD86 were reduced after exposure to 3O-C12 (Figure 2C). The maturation marker CD80 was downregulated on spDCs exposed to 3O-C12 (Figure 2D). It was reported that the hyporesponse of DCs to TLR stimulation may involve the Th2 response (Gao et al., 2013). Here, we provide evidence that 3O-C12 could inhibit LPS-induced BMDC activation, as the surface markers ICAM1, CD40, CD80, and CD86 were downregulated and cytokine IL-6 and tumor necrosis factor (TNF)-α production was reduced (Figure 2E). The effect of 3O-C12 was similar on both spDCs and BMDCs, namely, a reduced expression of CD80 after LPS stimulation (Figure 2F). However, the quorum sensing molecules C4, C6, and C12 neither inhibited DC maturation nor blocked LPS-induced CD80 expression (Figure 2G). Furthermore, we investigated whether other TLR pathways could be blocked by 3O-C12. First, we evaluated the TLR expression level during BMDC development. TLR-3, TLR-4, and TLR-7 were upregulated and TLR-5 was downregulated on day 6 (Figure S2A). 3O-C12 inhibited TLR ligands 1–7- and TLR ligand 9-induced DC activation (Figure 3A). High-dose exposure to 3O-C12 caused DC apoptosis, whereas lower doses (5 and 10 μM) did not (Figure 3B). A high dose of 3O-C12 (25 μM) induced B and T cell apoptosis *in vitro* (Figure S2B), consistent with a previous study (Song et al., 2019). We further used the multiple cytokine detection system MILLIPLEX Multiplex Assays, which demonstrated that 3O-C12 changed chemokine expression. The chemokine CCL22 was secreted mainly by DCs, and its expression depended on nuclear factor (NF)-κB activation and was significantly downregulated (Figure 3C). In contrast, IL-20, IL-27, IL-23, and TIMP-1 expression levels were not changed (Figure 3C). Moreover, 3O-C12 could block NF-κB pathway activation in DCs (Figure 3D), consistent with a previous report (Kravchenko et al., 2008). Downregulation of CCL22 by 3O-C12 may be due to the reduced activation of the NF-κB pathway in DCs. Collectively, these results suggest that 3O-C12 changes DC phenotype by inhibiting TLR signaling and activating a RA response.

### The Specific Chemical Structure of AHLs Determines the RARE Response in DCs

**Figure 4:**
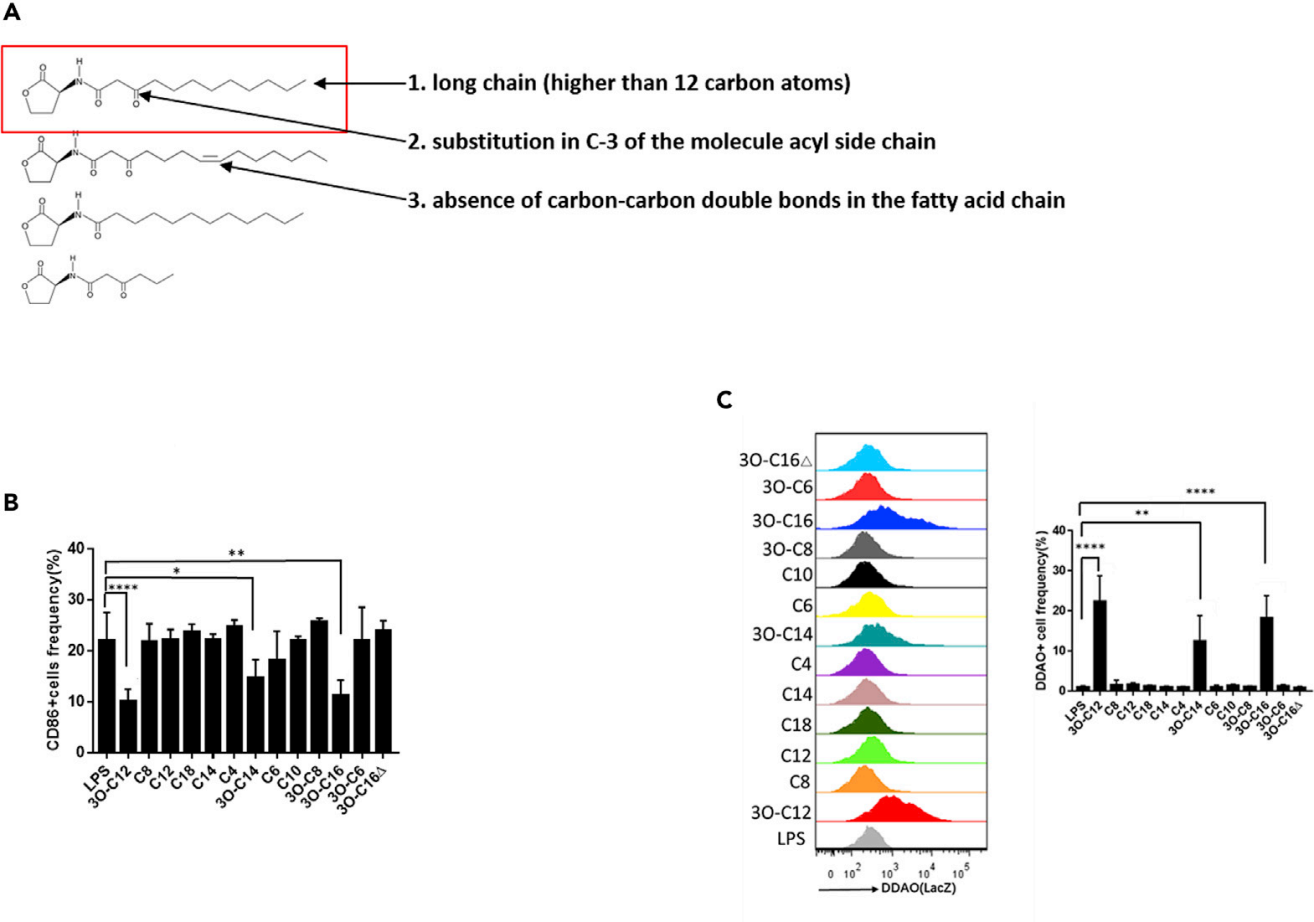
The Chemical Structure of AHLs Determines the RARE Response (A) The RARE response depends on the chemical structure of AHLs with three characteristics. (B) BMDCs (5–7 days) cultured from C57BL/6 mice were stimulated with LPS (100 ng/mL) with or without AHLs (10 μM) overnight. CD86-positive cells were estimated by flow cytometry. Data are representative of three independent experiments (n = 5–7). (C) BMDCs (5–7 days) cultured from RA reporter mice were stimulated with AHLs (10 μM) or LPS (100 ng/mL) for 1–2 h and incubated with DDAOG (10 μM) for 1–2 h. DDAO fluorescence was analyzed by flow cytometry. Data are representative of three independent experiments (n = 5–7). Data are presented as mean ± SD; p values were calculated using the two-tailed Student's t-test. ∗p < 0.05, ∗∗p < 0.01, ∗∗∗∗p < 0.0001; n.s., not significant.

The quorum sensing molecule AHL family includes numerous chemical molecules that differ in their R-group side-chain length. Chain lengths vary from 4 to 18 carbon atoms and in the substitution of a carbonyl at the third carbon. To pinpoint which chemical structure of AHL could activate RA signaling in DCs, 13 AHLs were screened (Table S1). Only the long chain (R-group side-chain lengths of more than 12 carbon atoms) and C-3 substitution in the acyl side chain exhibited the ability to activate RA signaling (Figures 4A and 4C); the only exception was 3O-C16△ with one carbon-carbon double bond in the fatty acid chain. The result was correlated with DC inhibition and downregulation of CD86 expression by these AHLs (Figure 4B). These data identified the chemical structure of AHLs that is responsible for activating RA signaling in DCs (Figures 4A and 4C).

### 3O-C12 Activates the DC RARE Response through the Inhibition of AKT Signaling

**Figure 5:**
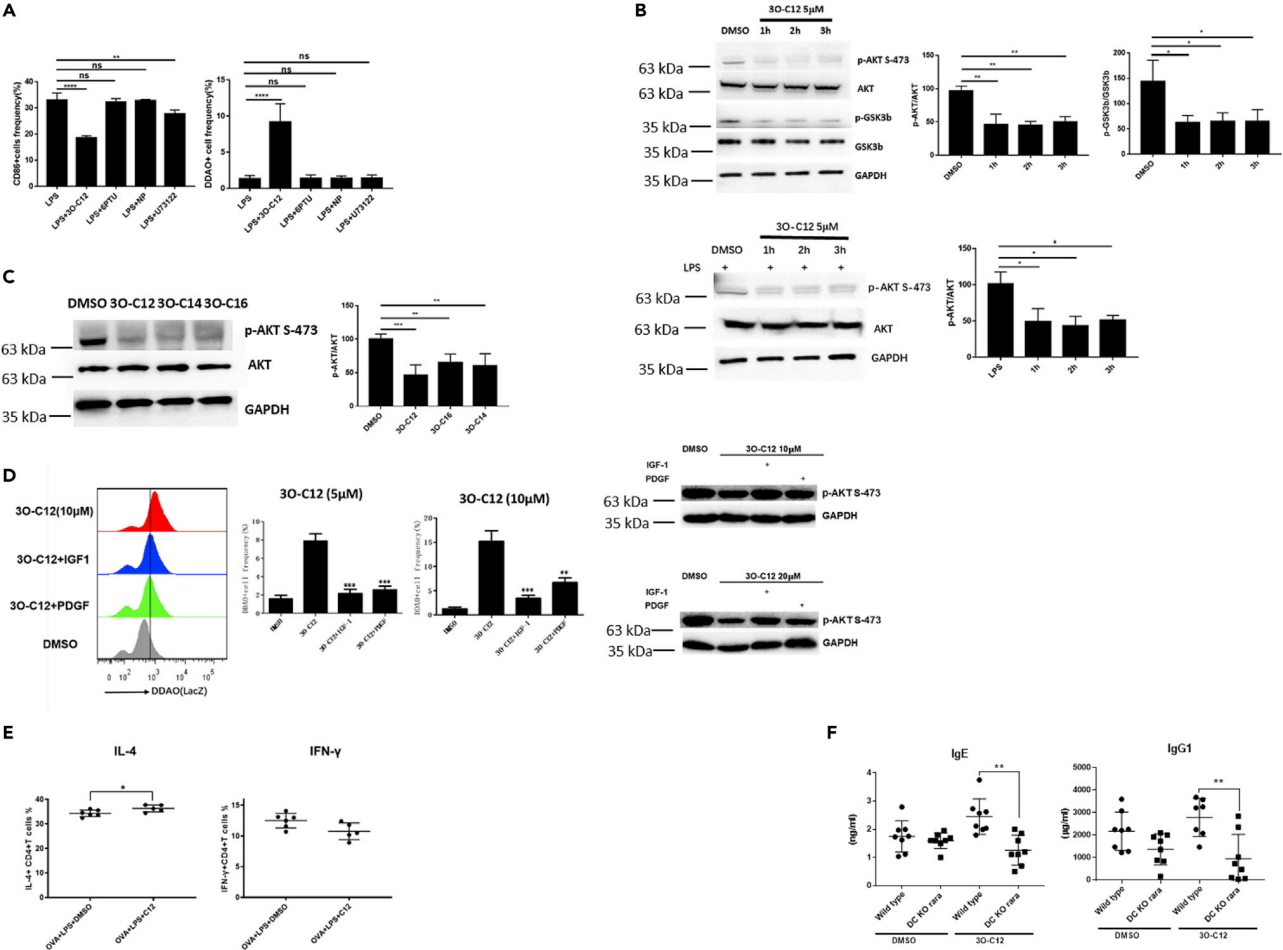
3O-C12 Activates the RARE Response by Disrupting AKT Signaling (A) BMDCs (5–7 days) cultured from RA reporter mice were stimulated with LPS alone (100 ng/mL) or LPS plus 3O-C12 (33 μM), 6PTU (100 uM), NP (100 uM), or U73122 (10 μM) overnight and incubated with DDAOG (10 μM) for 1–2 h. DDAO fluorescence and CD86 expression were analyzed by flow cytometry. Data are representative of three independent experiments. (B and C) (B) BMDCs (5–7 days) cultured from C57BL/6 mice were incubated with 3O-C12 (10 μM) for 1–3 h or (C) LPS plus 3O-C12. Cells were collected and lysed, and AKT phosphorylation was determined by western blot. Results are representative of three independent experiments. (D) BMDCs (5–7 days) cultured from RA reporter mice were stimulated with 3O-C12 (5 μM and 10 μM) or 3O-C12 plus IGF-1 (200 ng/mL) and PDGF (40 ng/mL) for 1–2 h and incubated with DDAOG (10 μM) for 1–2 h. DDAO fluorescence was analyzed by flow cytometry. AKT phosphorylation was determined by western blot. Data are representative of three independent experiments. (E) BMDCs (5–7 days) cultured from C57BL/6 mice were loaded with OVA (100 μg/mL), incubated with LPS, or LPS+3O-C12 (10 μM) for 1–3 h; naive OT-II cells were added and co-cultured for 5 days. IL4- and IFN-γ-positive cells were determined by flow cytometry. Data are representative of three independent experiments. (F) 6- to 8-week-old male and female CD11ccre^+^Rara^fl/fl^ mice (DC KO Rara) and littermate control CD11ccre^-^Rara^fl/fl^ mice (wild-type) were subcutaneously immunized with OVA (50 μg/mice) + LPS (0.5 μg/mice) with or without 3O-C12 (250 μg/mouse) every week for 3 weeks. Mouse serum was collected, and OVA-specific IgE and IgG1 were analyzed by ELISA. Results are representative of three independent experiments. Data are presented as mean ± SD; p values were calculated using the two-tailed Student's t test. ∗p < 0.05, ∗∗p < 0.01, ∗∗∗p < 0.001, ∗∗∗∗p < 0.0001; n.s., not significant.

To investigate the mechanism by which 3O-C12 activates the DC RARE response, we assessed the T2R38 taste receptor previously reported as a sensing receptor for 3O-C12. We investigated agonist binding to the T2R38 receptor as well as induced phospholipase C activation (Lee et al., 2012). Activating T2R38 with the agonists 6PTU (6-n-propylthiouracil) and NP (N-phenylthiourea) or inhibiting phospholipase C activation with U73122 neither changed the RARE response nor downregulated CD86 in DCs (Figure 5A). This indicated that T2R38 is not linked to the DC RARE response. AKT signaling is indispensable for TLR activation (Joung et al., 2011; Lee et al., 2003; Park et al., 2006). AKT also suppresses the transactivation of Rara in a subset of non-small cell lung cancer cells (Srinivas et al., 2006). We reasoned that 3O-C12 may regulate AKT signaling involved in the DC RARE response. We found that 3O-C12 could quench AKT activation in DCs with decreased phosphorylation of AKT (s-473) and GSK-3b, a downstream target that is phosphorylated by AKT (Figure 5B). Other AHLs such as 3O-C14 and 3O-C16, which could stimulate a RARE response in DCs, also exhibited the ability to quench AKT phosphorylation (Figure 5C). Furthermore, activation of the PI3K pathway through insulin-like growth factor 1 (IGF-1) and platelet-derived growth factor (PDGF) abolished the 3O-C12-activated DC RARE response (Figure 5D). These data indicate that 3O-C12 activates the DC RARE response through the inhibition of AKT signaling.

### 3O-C12-Induced Th2 Differentiation Depends on the Transcription Factor Rara

We next investigated whether 3O-C12 could induce DCs to prime CD4+ T cells toward Th2 differentiation. Isolated OT-II cells were co-cultured with DCs in the presence of the OVA antigen. After exposure to 3O-C12, DCs stimulated CD4+ T cells toward Th2 differentiation, as IL-4^+^ cells were upregulated (Figure 5E) and produced high levels of IL-5 and IL-13 (Figure S2C).

To confirm if a lack of RA signaling in DCs impairs IgE and IgG1 production *in vivo*, we established DC-specific Rara gene knockout (KO) mice (CD11c^cre^Rara^fl/fl^), as Rara is the dominant RA signaling transcription factor in DCs (Galvin et al., 2013). After immunization with OVA plus LPS and 3O-C12, we found that the DC-specific KO of the Rara gene (DC KO rara) significantly reduced OVA-specific IgE and IgG1 production in mice (Figures 5F and S2E). However, DC KO rara did not change basal immunoglobulin levels (Figure S2F). Furthermore, DC KO rara impaired CD4+ T cell Th2 differentiation in 3O-C12-activated BMDCs *in vitro* and *in vivo* (Figures S2D and S2H). Collectively, these data indicate that the quorum sensing molecule 3O-C12 boosts the allergic type 2 immune response by activating RA signaling in DCs.

**Figure 6:**
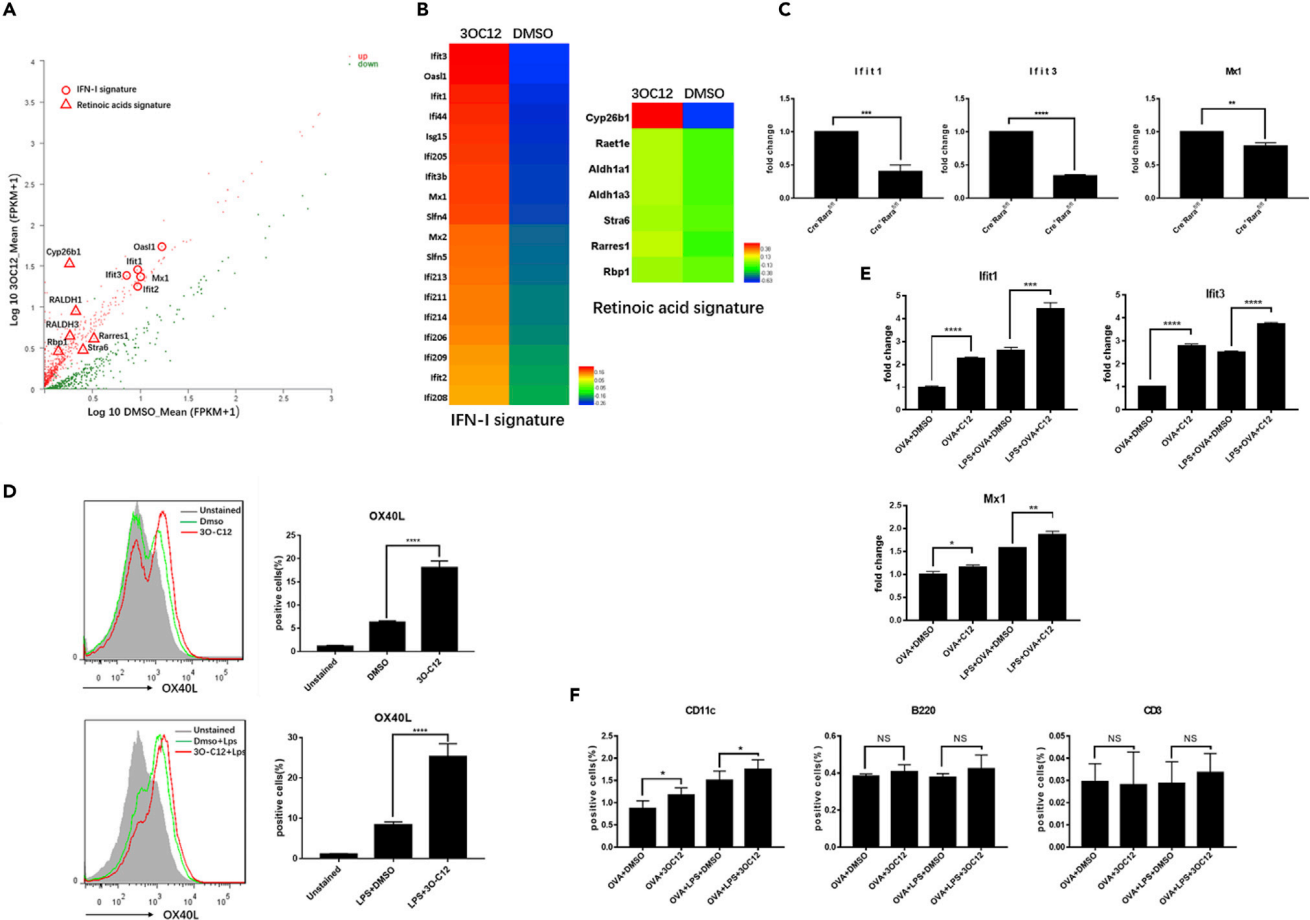
3O-C12 Stimulates the IFN-I Signature and OX40L (A and B) RNA sequencing analysis of the gene expression of 3O-C12-activated DCs. (A) Scatterplot of gene expression of FPKM values. (B) Heatmap of FPKM values, IFN-I signature, and RA signature. (C) The 3O-C12-stimulated IFN-I signature depends on Rara. Gene expression levels were examined by qPCR. Results are representative of two independent experiments. (D) Fluorescence-activated cell sorting analysis of OX40L in 3O-C12-activated DCs (upper panel) and 3O-C12+LPS-activated DCs (lower panel). Results are representative of four independent experiments. (E) 3O-C12 stimulates expression of the IFN-I signature in DCs *in vivo*. Results are representative of three independent experiments. (F) 3O-C12 upregulates OX40L in DCs *in vivo*. Results are representative of two independent experiments (n = 5–7). p values were calculated using the two-tailed Student's t test. ∗p < 0.05, ∗∗p < 0.01, ∗∗∗p < 0.001, ∗∗∗∗p < 0.0001; n.s., not significant.

To gain further insight into the mechanism of 3O-C12-activated DCs and to identify the cellular targets of 3O-C12 that are involved in CD4+T cell Th2 differentiation, we isolated mRNA from 3O-C12-activated and control DCs and subjected it to Illumina transcriptome sequencing. Transcripts with p value <0.05 between the two groups were graphed by functions. Among the genes differentially expressed, RA signatures, including Cyp26b1, RALDH1 (Aldh1a1), and RALDH3 (Aldh1a3), were upregulated in 3O-C12-activated DCs (Figures 6A and 6B), consistent with the result that 3O-C12 could stimulate a RARE response in DCs. Unexpectedly, the type I interferon (IFN-I) signatures, including Ifit1, Mx1, and Oasl1, were upregulated in 3O-C12-activated DCs (Figures 6A and 6B). The IFN-I signature has been well defined as a critical player in DCs for the initiation of the Th2 response (Connor et al., 2017; Janss et al., 2016; Webb et al., 2017). The IFN-I signature result was confirmed by qPCR (Figure S3A). Chemokines and chemokine receptors were also altered in 3O-C12-stimulated DCs (Figure S3B). Next, we confirmed that the 3O-C12-induced RA and IFN-I signatures were dependent on the transcription factor Rara, as 3O-C12 was unable to upregulate these two signatures in BMDCs from DC Rara KO mice (Figures S3C and 6C).

OX4OL has been indicated as a critical molecule in Th2 cell induction primed by DCs. Here, we found that 3O-C12 with or without LPS could enhance the expression of OX4OL in DCs (Figure 6D). Immunization of mice with OVA/3O-C12 could stimulate OX40L expression on DCs *in vivo*, but not on B or T cells in the drain lymph node (Figure 6F). Furthermore, we confirmed that 3O-C12 upregulated the IFN-I signature in DCs *in vivo* (Figure 6E), suggesting that 3O-C12 specifically targets DCs.

We observed that LPS could slightly enhance 3O-C12-induced IgE and IgG1 production (Figure 1B). LPS-activated DCs favor Th1 induction by the inhibition of Th2 differentiation via the TNF-α/IL-12 circuit (Bachus et al., 2019). However, in some cases, it has been suggested that LPS can contribute to the severity of Th2 lung inflammation and food allergies (Michel et al., 1996; Rodriguez et al., 2017), which may involve OX40L (Duan et al., 2008) and IFN-I (Pollara et al., 2006). To clarify the synergic role of LPS in the 3O-C12-induced type 2 immune response, we isolated mRNA from LPS- and LPS/3O-C12-activated DCs, which was then subjected to Illumina transcriptome sequencing. Transcripts with p values <0.05 between the two groups were graphed by functions. Compared with LPS alone, LPS plus 3O-C12 upregulated the IFN-I signature, RA signature, and chemokines/chemokine receptors (Figures S4A−S4C), which suggests a vital role of 3O-C12 in the upregulation of these genes. Furthermore, LPS could enhance the effect of 3O-C12 on the IFN-I signature, but mitigated the RA signature compared with 3O-C12 alone (Figure S4E). Fn14 (TNFRSF12A or TweakR), previously reported as a Th2-driven factor (Son et al., 2013), was also upregulated by 3O-C12, observed with RNA sequencing (RNA-seq) (Figure S3C) or qPCR (Figure S4D). Moreover, 3O-C12 enhances the expression of OX40L while reducing the production of IL-12 in LPS-stimulated DCs (Figure S4F). Next, we tested whether 3O-C12 can upregulate the transcriptional factor c-Maf, previously reported as an inhibitor of IL-12 (Homma et al., 2007). However, c-Maf was not changed by 3O-C12 (Figure S4G), which suggests that the inhibitory effect of 3O-C12 on IL-12 is not due to c-Maf induction. Collectively, 3O-C12 stimulates DC upregulation of Th2-driving genes (type I-IFN and OX40L).

## Discussion

The high colonization rate of *P. aeruginosa* in allergic patients implies that this bacterium may provoke allergies. In this study, we suggest that structure-specific quorum sensing AHL molecules promote OVA-specific IgE and IgG1 production *in vivo* by activating the RARE response in DCs. Cell-specific deletion of RA transcriptional factor Rara in DCs diminishes AHL-induced IgE and IgG1 production. Moreover, we further found that AHLs activate the RARE response by inhibiting AKT phosphorylation. DC exposure to AHLs inhibits the NF-κB pathway and quenches TLR activation.

Quorum sensing is a process of communication among bacteria that allows the coordination of group behavior based on cell density. During bacterial signaling, AHLs are produced within the bacterial cell and released into the environment. Bacteria-derived molecules like TLR and short-chain fatty acids have been demonstrated to inhibit antigenic IgE production, which reflects the basis of the “hygiene hypothesis.” In this study, administration of 3O-C12 increased allergic lung inflammation in a mouse model of asthma. The polarization of Th1, Th2, and Th17 cells was observed in the lung, which differs from the effect of 3O-C12 on CD4^+^T cell Th2 polarization *in vitro*. These interesting differences may be due to the different mouse models *in vitro* and *in vivo*. Intranasal transfer of OVA-loaded, 3O-C12 activated BMDC induced OVA-specific IgE, but not IgG1. This result may reflect the different effect of AHLs on DCs *in vitro* and *in vivo*. Our findings provide a mechanism by which the bacterial quorum sensing molecules AHLs modulate DC signaling, which facilitates IgE and IgG1 production.

RA is synthesized from retinol via two enzymatic reactions, involving first, reversible oxidation of retinol to retinal and then a second oxidation, irreversible metabolism into RA via retinal dehydrogenases (RALDH). DCs from gut-associated lymphoid tissue (GALT) and the draining lymph nodes of the skin and lung express RALDH, and have the capacity to synthesize RA. In addition to DCs, several nonhematopoietic lineages within GALT, such as epithelia and stromal cells share the capacity to synthesize RA. RAs are essential for immune system development (Zhang et al., 2016) and maintenance of host defense (Hall et al., 2011b). RAs have been demonstrated to stimulate CD4+ T cells for Th2 differentiation. Here, we found that AHLs could stimulate the type 2 immune reaction by inducing the RARE response in DCs. DC-specific KO of Rara reverses the effect of AHLs on OVA-specific IgE and IgG1 production. Cre recombinase is widely used to precisely manipulate genes and chromosomes, but it can also display off-target activity. In this study, CD11c cre^−^ Rara^fl/fl^ mice were used as littermate controls, and we did not add CD11c cre^+^ RARA^wt/wt^ mice as controls.

The AKT pathway is indispensable for TLR activation in DCs. In this study, we illustrated that AHLs inhibit TLR activation, but activate the RARE response by quenching AKT phosphorylation in DCs. Furthermore, we demonstrated that only a long chain (R-group side-chain lengths with more than 12 carbon atoms), absence of carbon-carbon double bonds in the fatty acid chain, and C-3 substitution in the acyl side chain have the ability to activate RA signaling. Stimulation of DCs with IGF-1 and PDGF, two potent agonists of AKT, eliminated the stimulative effect of AHLs on RARE. Our study links AKT signaling and the RARE response with the AHL-induced type 2 immune response. However, the mechanisms of how specific chemical structures of AHLs block AKT phosphorylation need to be further investigated.

AHLs stimulated DCs to be hyporesponsive to TLR. RNA-seq analysis revealed that AHLs induced the IFN-I and RA signatures. This phenotype of AHL-activated DCs resembles a previously reported DC subset involved in Th2 priming (Gao et al., 2013), with a characteristic reduced response to TLR and high expression of Mx1 (IFN-I signature) and Aldh1a2 (RA signature). Furthermore, we confirmed that AHL-stimulated IFN-I and RA signatures both depended on the Rara transcriptional factor. Although the role of Rara in effector CD4+ T cells has been studied (Hall et al., 2011a), it is unclear if it modulates the phenotype of DCs for Th1 or Th2 induction. In this study, we provide evidence that DC-intrinsic Rara can act as a Th2 induction factor.

IL-12 production in DCs induces the differentiation of naive CD4+ T cells to become IFN-γ-producing Th1 cells (Trinchieri, 1994). The impaired production of IL-12 could induce DCs to prime Th2 cells, which may depend on TNF-α. OX40L has been well described as a principal factor in DCs for the induction of Th2 cells. Here, we provide evidence that AHLs upregulate OX40L but downregulate IL-12 in DCs. However, this phenotype may not only depend on Rara but also depend on the inhibitory role of AHLs on TNF-α.

Collectively, our research reveals that the quorum sensing molecules AHLs boost OVA-specific IgE and IgG1 production via AKT-Rara interactions in DCs. AHLs may change DC phenotype as Th2 cells prime DCs (DC_Th2_) with the following features: (1) hyporesponsiveness to TLR and (2) upregulated IFN-I signature, RA signature, and OX40L (Figure S5). Our research identifies the effect of a bacterial component on an allergic response and adds insight into the process of bacteria-induced IgE and IgG1 production. We have also demonstrated that when clinicians use specific bacterial strains to regulate allergic diseases (Niers et al., 2007; Toh et al., 2012), they should keep in mind the potential release of the quorum sensing molecules AHLs.

### Limitations of the Study

There are several limitations of this study. First, we did not use the AHLs KO strain of *P. aeruginosa* in this study. Second, CD11c cre- Rara^fl/fl^ mice were used as littermate controls, and we did not add CD11c cre + RARA^wt/wt^ mice as controls.

### Resource Availability

#### Lead Contact

Further information should be directed to the Lead Contact, Zongde Zhang (zongdez@swmu.edu.cn).

#### Materials Availability

Materials in this study are available from the Lead Contact upon reasonable request.

#### Data and Code Availability

Raw sequencing data were deposited in the NCBI Sequence Read Archive (SRA) under Project Accession: PRJNA631431.

## Methods

All methods can be found in the accompanying Transparent Methods supplemental file.

## Supplemental Information

Document S1. Transparent Methods, Figures S1–S5, and Table S1
