# Supplementary material for: Bacterial Quorum Sensing Molecules Promote Allergic Airway Inflammation by Activating the Retinoic Acid Response
Source: iScience. 2020 Jun 20;23(7):101288. doi: 10.1016/j.isci.2020.101288 (PMC7334417; doi:10.1016/j.isci.2020.101288)
Supplement: Document S1. Transparent Methods, Figures S1–S5, and Table S1 [file mmc1.pdf]

iScience, Volume *23*

Supplemental Information

Bacterial Quorum Sensing Molecules Promote Allergic Airway Inflammation by Activating the Retinoic Acid Response

Renlan Wu, Xingjie Li, Ning Ma, Xiufeng Jin, Xiefang Yuan, Chen Qu, Hongmei Tang, Zhigang Liu, and Zongde Zhang

Fig. S1

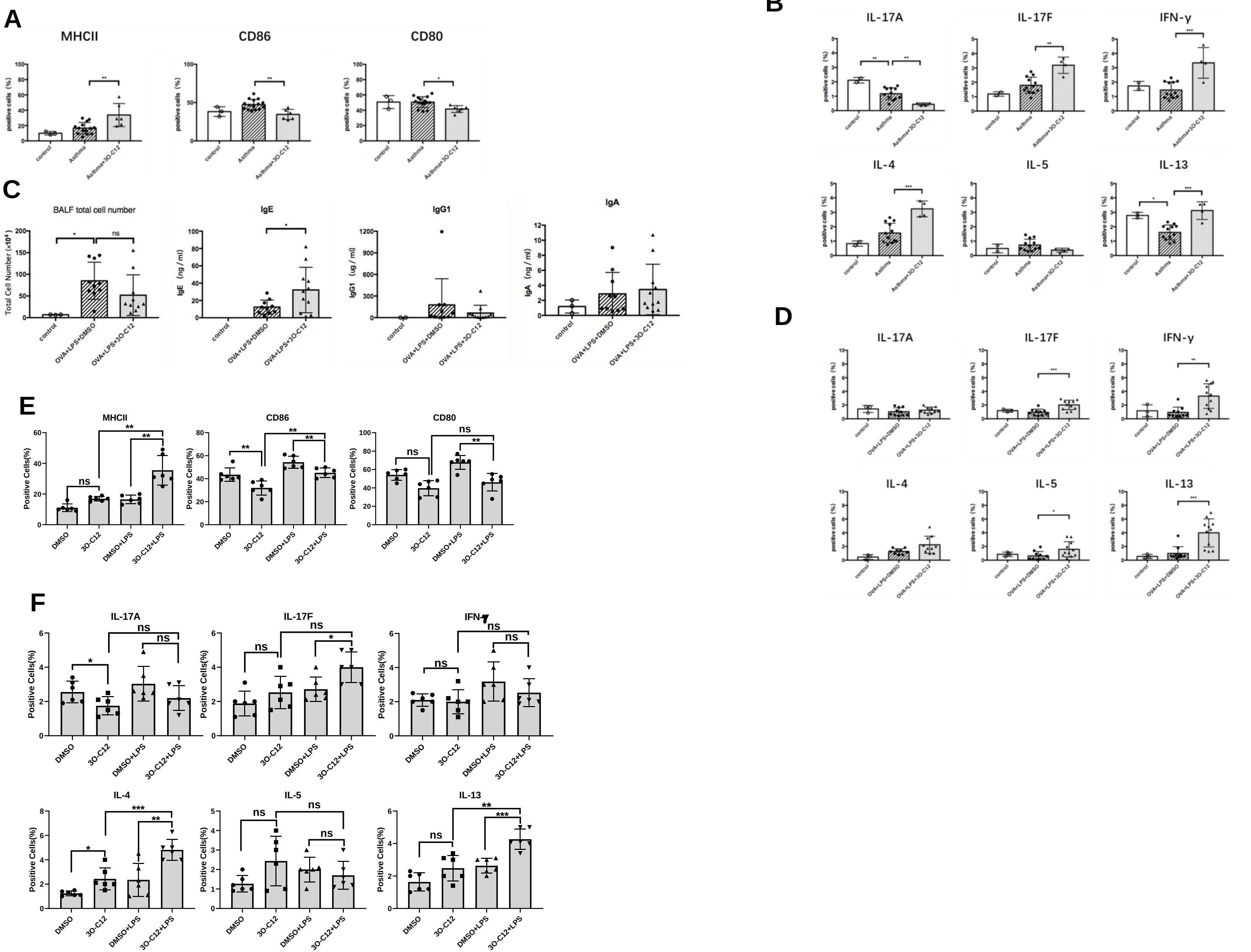
A
MHCII
CD86
CD80
positive cells (%)
control
Asthma
Asthma+3O-C12
B
IL-17A
IL-17F
IFN-γ
IL-4
IL-5
IL-13
C
BALF total cell number
Total Cell Number (×10⁴)
IgE
IgE (ng / ml)
IgG1
IgG1 (ug / ml)
IgA
IgA (ng / ml)
OVA+LPS+DMSO
OVA+LPS+3O-C12
D
E
Positive Cells(%)
DMSO
3O-C12
DMSO+LPS
3O-C12+LPS
F
IFN-[illegible]

Fig. S2

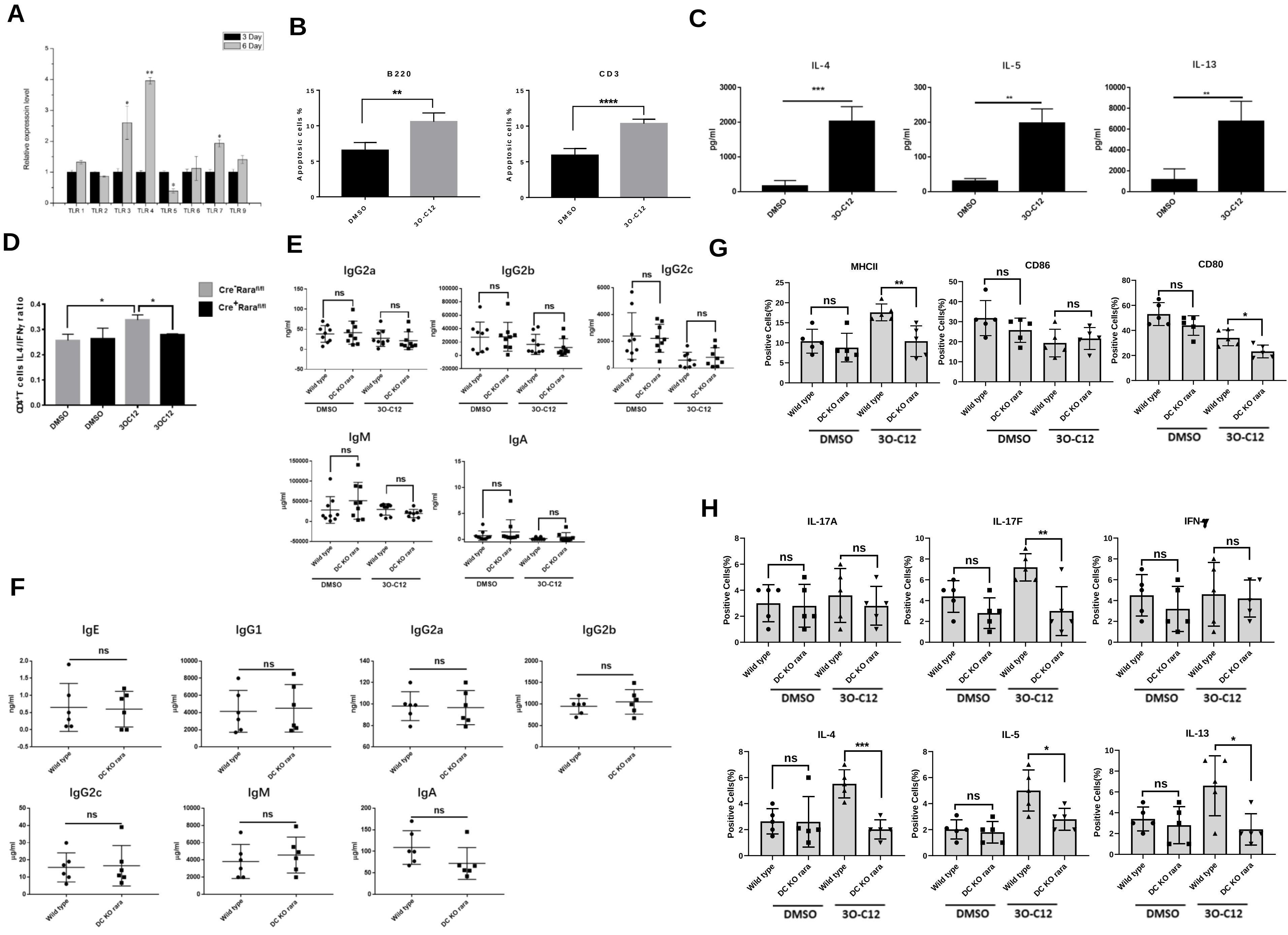

A
3 Day
6 Day
Relative expressoin level
TLR 1
TLR 2
TLR 3
TLR 4
TLR 5
TLR 6
TLR 7
TLR 9
B
B220
CD3
Apoptosic cells %
DMSO
3O-C12
C
IL-4
IL-5
IL-13
pg/ml
DMSO
3O-C12
D
Cre⁻Rara fl/fl
Cre⁺Rara fl/fl
CD4⁺T cells IL4/IFNγ ratio
DMSO
3OC12
E
IgG2a
IgG2b
IgG2c
IgM
IgA
ng/ml
μg/ml
Wild type
DC KO rara
DMSO
3O-C12
ns
F
IgE
IgG1
IgG2a
IgG2b
IgG2c
IgM
IgA
G
MHCII
CD86
CD80
Positive Cells(%)
H
IL-17A
IL-17F
IFN-γ
IL-4
IL-5
IL-13
Positive Cells(%)

Fig. S3

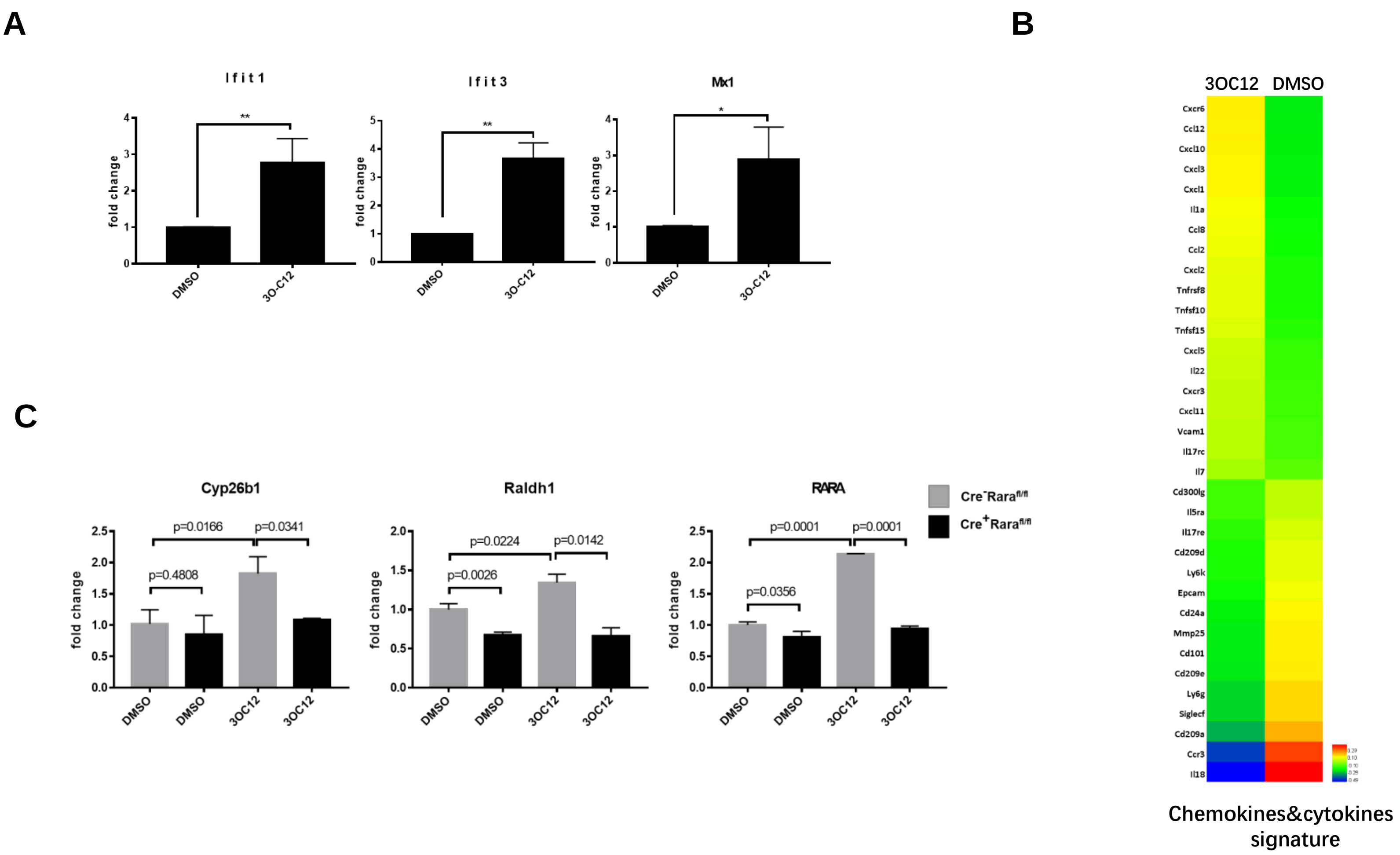
A
Ifit1
Ifit3
Mx1
fold change
DMSO
3O-C12
**
**
*
B
3OC12
DMSO
Cxcr6
Ccl12
Cxcl10
Cxcl3
Cxcl1
Il1a
Ccl8
Ccl2
Cxcl2
Tnfrsf8
Tnfsf10
Tnfsf15
Cxcl5
Il22
Cxcr3
Cxcl11
Vcam1
Il17rc
Il7
Cd300lg
Il5ra
Il17re
Cd209d
Ly6k
Epcam
Cd24a
Mmp25
Cd101
Cd209e
Ly6g
Siglecf
Cd209a
Ccr3
Il18
Chemokines&cytokines signature
C
Cyp26b1
Raldh1
RARA
fold change
p=0.0166
p=0.0341
p=0.4808
p=0.0224
p=0.0142
p=0.0026
p=0.0001
p=0.0001
p=0.0356
DMSO
3OC12
Cre⁻Rara fl/fl
Cre⁺Rara fl/fl

Fig. S4

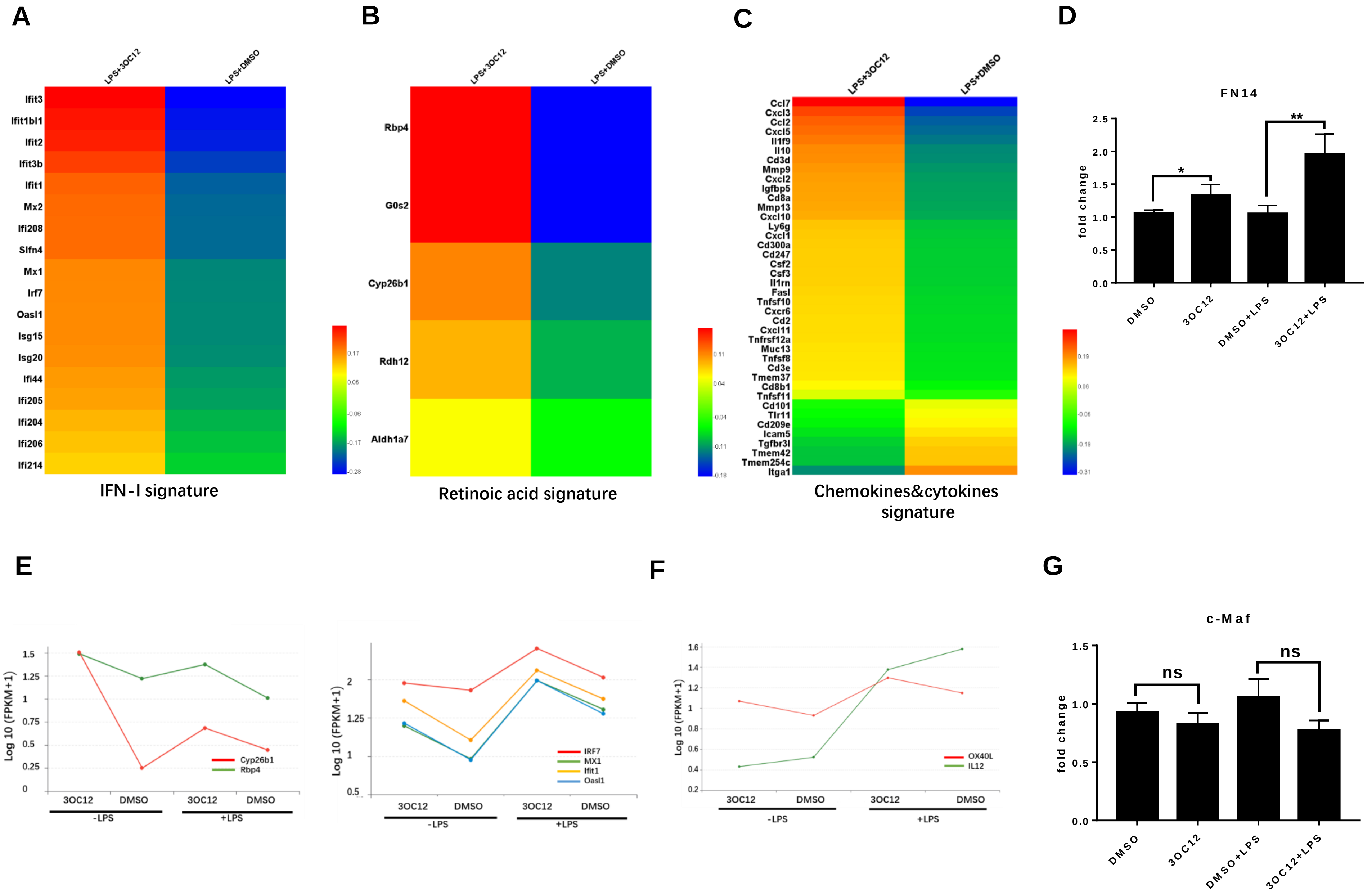

A
LPS+3OC12
LPS+DMSO
Ifit3
Ifit1bl1
Ifit2
Ifit3b
Ifit1
Mx2
Ifi208
Slfn4
Mx1
Irf7
Oasl1
Isg15
Isg20
Ifi44
Ifi205
Ifi204
Ifi206
Ifi214
IFN-I signature
B
Rbp4
G0s2
Cyp26b1
Rdh12
Aldh1a7
Retinoic acid signature
C
Ccl7
Cxcl3
Ccl2
Cxcl5
Il1f9
Il10
Cd3d
Mmp9
Cxcl2
Igfbp5
Cd8a
Mmp13
Cxcl10
Ly6g
Cxcl1
Cd300a
Cd247
Csf2
Csf3
Il1rn
Fasl
Tnfsf10
Cxcr6
Cd2
Cxcl11
Tnfrsf12a
Muc13
Tnfsf8
Cd3e
Tmem37
Cd8b1
Tnfsf11
Cd101
Tlr11
Cd209e
Icam5
Tgfbr3l
Tmem42
Tmem254c
Itga1
Chemokines&cytokines signature
D
FN14
fold change
DMSO
3OC12
DMSO+LPS
3OC12+LPS
E
Log 10 (FPKM+1)
Cyp26b1
Rbp4
IRF7
MX1
Ifit1
Oasl1
3OC12
DMSO
-LPS
+LPS
F
OX40L
IL12
G
c-Maf
ns

Fig. S5

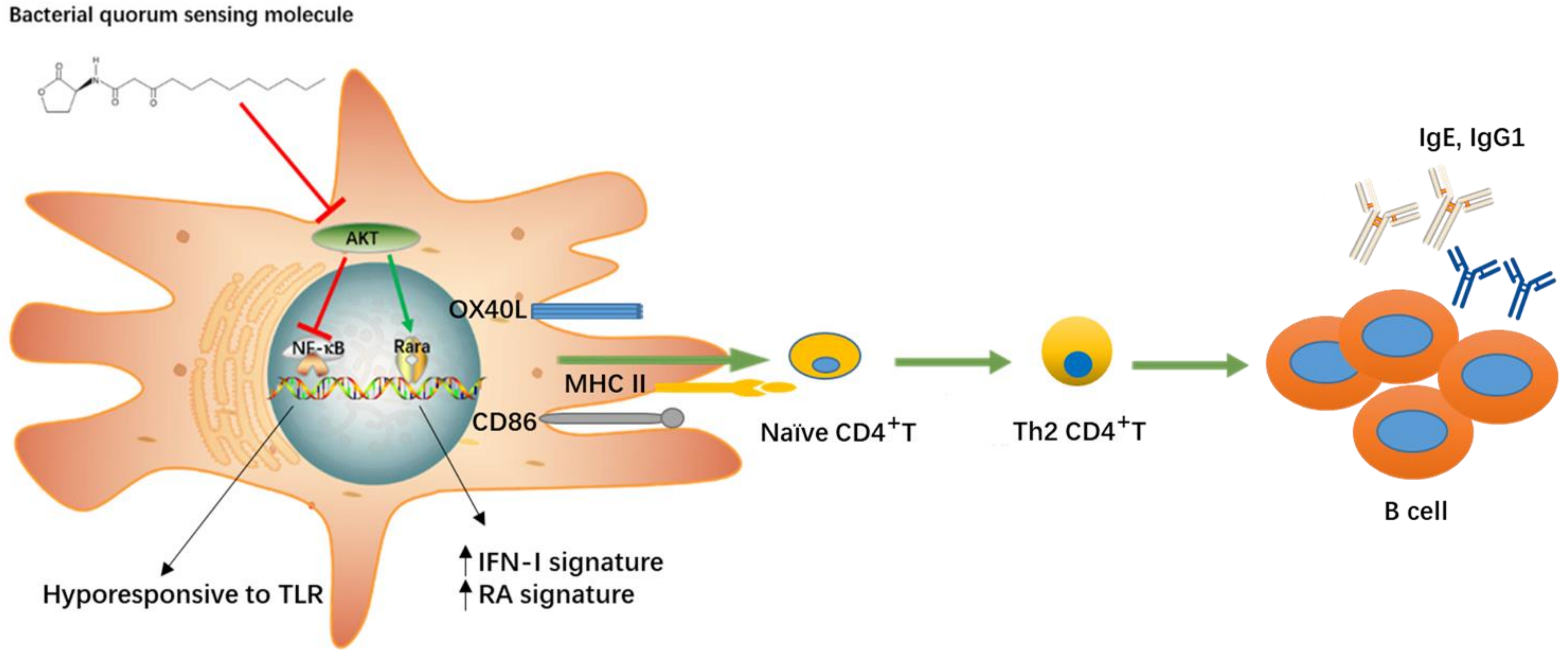
Bacterial quorum sensing molecule
AKT
NF-κB
Rara
OX40L
MHC II
CD86
Hyporesponsive to TLR
IFN-I signature
RA signature
Naïve CD4+T
Th2 CD4+T
IgE, IgG1
B cell

Table S1

Item	Name	Synonyms	Chemical structure
10007895	N-3-oxo-dodecanoyl-L-Homoserine lactone	3O-C12	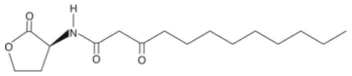
10011199	N-octanoyl-L-Homoserine lactone	C8	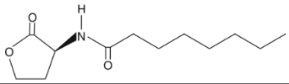
10011203	N-dodecanoyl-L-Homoserine lactone	C12	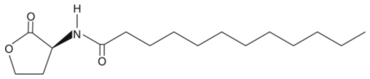
13209	N-octadecanoyl-L-Homoserine lactone	C18	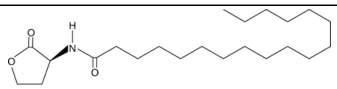
10011200	N-tetradecanoyl-L-Homoserine lactone	C14	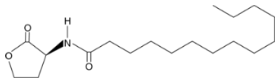
10007898	N-butyryl-L-Homoserine lactone	C4	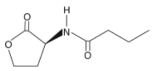
13063	N-3-oxo-tetradecanoyl-L-Homoserine lactone	3O-C14	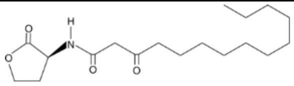
10007896	N-hexanoyl-L-Homoserine lactone	C6	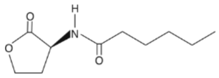
10011201	N-decanoyl-L-Homoserine lactone	C10	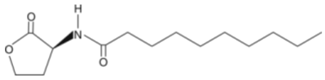
10011206	N-3-oxo-octanoyl-L-Homoserine lactone	3O-C8	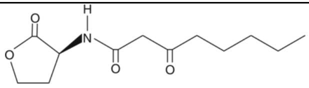
13062	N-3-oxo-hexadecanoyl-L-Homoserine lactone	3O-C16	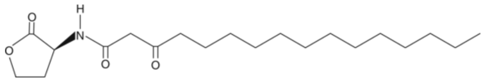
10011207	N-(β -ketocaproyl)-L-Homoserine lactone	3O-C6	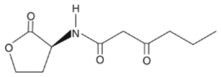
10011238	N-3-oxo-hexadec-11(Z)-enoyl-L-Homoserine lactone	3O-C16Δ	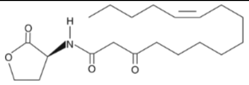

Table S1. Chemical structure of AHLs. Related to Figure 4.

Figure S1. 3O-C12 enhances allergic lung inflammation. Related to Figure 1.

(A) Balb/c mice were divided into three groups: control (n = 4), asthma (n = 15), and asthma + 3O-C12 (n = 6). The mice were intraperitoneally (i.p.) injected on day 1 and day 13 with OVA (50 μg) and aluminum hydroxide (2 mg). On day 20 and 22, the mice were intranasally (i.n.) challenged with OVA (40 μg) and i.p. administered with or without 3O-C12 (500 μg). On day 25, 27, and 29, the mice were i.n. challenged with OVA (40 μg) and i.n. administered with or without 3O-C12 (50 μg). One day after the last OVA challenge, mice were sacrificed. Expression of DC surface markers **(A)** and cytokines of CD4$^+$T **(B)** from spleen were determined by flow cytometry. Data are representative of three independent experiments

(C) BMDCs from C57BL/6 mice were stimulated by OVA (200 μg/ml) and LPS (100 ng/ml) overnight, with or without 3O-C12 (10 μM). BMDCs were transferred intranasally (i.n.) to recipients (C57BL/6 mice) (n = 3 in control group; n = 10 in OVA + LPS + DMSO group; n = 11 in OVA + LPS + 3O-C12 group) on days 1 and 12. Each recipient was challenged by 40 μg OVA i.n. on days 13, 14, and 15. One day after the last OVA challenge, mice were sacrificed and assessed for bronchoalveolar lavage fluid (BALF) total cell number **(C)**, serum immunoglobulins **(C)**, and CD4$^+$ T cell cytokine production from the spleen **(D)**.

(E−F) Wild-type C57BL/6 mice were subcutaneously immunized with OVA (50 μg/mouse) + LPS (0.5 μg/mouse) with or without 3O-C12 (250 μg/mouse) every week for three weeks; incomplete Freund adjuvant was used as a vehicle. Expression of DC surface markers **(E)** and cytokines of CD4$^+$T **(F)** from spleen were determined by flow cytometry. Results are representative of three independent experiments.

Data are presented as mean ± SD; p-values were calculated using the two-tailed Student's t-test. *$p < 0.05$, **$p < 0.01$, ***$p < 0.001$, ****$p < 0.0001$, n.s.: not significant.

Figure S2. 3O-C12-activated DCs induced CD4+T Th2 differentiation that depended on Rara. Related to Figure 1 and Figure 5.

(A) Quantitative real-time PCR analysis of the TLR expression pattern of BMDCs cultured for 3 and 6 d.

(B) Spleen cells cultured from C57BL/6 mice were incubated with 3O-C12 (10 μM) for 12 h, annexin v-positive cells gated on B220 and CD3 were determined by flow cytometry.

(C) OT-II cells were activated by 3O-C12-conditioned DCs in the presence of OVA antigen for 5 d and restimulated with anti-CD3 and anti-CD28 antibody for cytokine analysis.

(D) BMDCs (5−7 d) cultured from CD11ccre$^+$Rara$^{\mathbf{fl/fl}}$ mice (Cre+Rara$^{fl/fl}$) and littermate control CD11ccre$^-$Rara$^{\mathbf{fl/fl}}$ mice (Cre-Rara$^{fl/fl}$), were loaded with OVA (100 μg/mL) and incubated with LPS or LPS+3O-C12 (10 μM) for 1−3 h. Naive OT-II cells were added and co-cultured for 5 d. IL-4- and IFN-γ-positive cells were determined by flow cytometry.

(E) CD11ccre$^+$Rara$^{\mathbf{fl/fl}}$ mice (DC KO Rara) and littermate control CD11ccre$^-$Rara$^{\mathbf{fl/fl}}$ mice (wild-type) were subcutaneously immunized with OVA (50 μg/mouse) + LPS (0.5 μg/mouse) with or without 3O-C12 (250 μg/mouse) every week for three weeks. Mouse serum was collected and OVA-specific immunoglobulins were analyzed by ELISA.

(F) Basal levels of immunoglobulins in CD11ccre$^+$Rara$^{\mathbf{fl/fl}}$ mice (DC KO Rara) and littmite control CD11ccre$^-$Rara$^{\mathbf{fl/fl}}$ mice (wild-type) were analyzed by ELISA.

(G−H) CD11ccre$^+$Rara$^{\mathbf{fl/fl}}$ mice (DC KO Rara) and littermate control CD11ccre$^-$Rara$^{\mathbf{fl/fl}}$ mice (wild-type) were

subcutaneously immunized with OVA (50 μg/mouse) + LPS (0.5 μg/mouse) with or without 3O-C12 (250 μg/mouse) every week for three weeks. Expression of DC surface markers **(G)** and cytokines of $CD4^{+}$T cells **(H)** from spleen were determined by flow cytometry.

Data are representative of three independent experiments. Data are presented as mean ± SD; p-values were calculated using the two-tailed Student's t-test. *p < 0.05, **p < 0.01, ***p < 0.001, ****p < 0.0001, n.s.: not significant.

Figure S3. 3O-C12 upregulated multiple genes in DCs. Related to Figure 6.

(A) The IFN-I signature stimulated by 3O-C12 was examined with Q-PCR.

(B) RNA-seq analysis of chemokines and chemokine receptor gene expression of 3O-C12-activated DCs. Heatmap of FPKM values.

(C) The RA signature stimulated by 3O-C12 was dependent on Rara.

Data of Q-PCR are representative of three independent experiments. Data are presented as mean ± SD; p-values were calculated using the two-tailed Student's t-test. *p < 0.05, **p < 0.01, ***p < 0.001, ****p < 0.0001, n.s.: not significant.

Figure S4. LPS enhances the 3O-C12-stimulated expression of the IFN-I signature and OX40L. Related to Figure 6.

(A−E) RNA-seq analysis of the gene expression of 3O-C12 + LPS-activated DCs.

(A−C) Heatmap of FPKM values.

(D) Q-PCR analysis of FN14 (TNFRSF12A) expression in DCs.

(E) The expression level of the IFN-I and RA signature in 3O-C12-activated and 3O-C12+LPS-activated DCs.

(F) The expression level of IL12 and OX40L in 3O-C12-activated and 3O-C12+LPS-activated DCs.

(G) Q-PCR analysis of c-Maf expression in DCs.

Data of Q-PCR are representative of three independent experiments. Data are presented as mean ± SD; p-values were calculated using the two-tailed Student's t-test. *p < 0.05, **p < 0.01, ***p < 0.001, ****p < 0.0001, n.s.: not significant.

Figure S5. Graphical representation of the phenotype of 3O-C12-activated DCs. Related to Figure 1 and Figure 6.

Materials and Methods

Ethics Statement

All animal experiments were performed in accordance with the recommendations outlined in the Guide for the Care and Use of Laboratory Animals. All research animal protocols were approved by the Institutional Animal Care and Use Committee at the Model Animal Research Center, Nanjing University (animal protocol number: AP#ZZD01)

Reagents

Acyl-homoserine lactone molecules (Table S1) were purchased from Cayman Chemical Company (Ann Arbor, USA), dissolved in DMSO, and stored at -20 ℃. TLR1-9 ligands used for stimulation of DCs were purchased from Invivogen. T2R38 receptor agonists 6PTU (6-n-propylthiouracil) and NP (N-Phenylthiourea), phospholipase C inhibitor U73122, and OVA were purchased from Sigma. DDAOG was purchased from Invitrogen and recombinant mouse IGF-1 and PDGF from R&D or Genscript.

Mice

The Tg(RARE-Hspa1b/lacZ)12Jrt/J strain was obtained from Jackson Laboratories and maintained as homozygotes to retain the reporter activity. $CD11c^{cre}$ and OT-II mice with a C57BL/6 background were purchased from Jackson Laboratories. $Rara^{fl/fl}$ mice were established in our lab as previously reported. C57BL/6 was purchased from the Nanjing Biomedical Research Institute of the Nanjing University. All research animal

protocols were approved by the Institutional Animal Care and Use Committee of the Model Animal Research Center, Nanjing University.

FACS and antibody

DCs were washed and suspended in FACS buffer (PBS, 2% FBS), incubated with fluorochrome-coupled agents for 30 min at 4 ℃, and then washed in FACS buffer. Data were obtained with the Accuri C6 Flow Cytometer (BD Biosciences) and analyzed by FlowJo software v9.6 (FlowJo, Ashland, USA). The following anti-murine antibodies were used for flow cytometric analysis: CD40 (HM40-3), CD80 (16-10A1), CD86 (GL1), ICAM1 (YN1/1.7.4), CD11c (N418), TCR V alpha 2 (B20.1), IL-4 (11B11), CD4 (RM4-5), B220 (RA3-6B2), CD3 (17A2) (all purchased from eBioscience), MHCII (M5/114.15.2), OX40L(RM134L), and IFN-ɣ (XMG1.2) (Biolegend).

BMDC culture and isolation of spleen DCs

DCs were generated from bone marrow (BM) cells obtained from 5–7-week-old, female and male RA reporter mice or C57BL/6 mice. Briefly, BM cells were flushed out from femurs and tibias. BM cells were cultured in complete culture medium (RPMI 1640 supplemented with 10% FBS, 25 mM HEPES, 5 mM 2-ME, and antibiotics; Hyclone) containing 10 ng/ml GM-CSF and 10 ng/ml IL-4 (Peprotech). Spleen DCs were isolated with magnetic-activated cell sorting (MACS) by EasySep™ Mouse CD11c Positive Selection Kit (stem cell), according to the manufacturer’s instructions.

Adoptive transfer of BMDCs

Bone marrow (BM) cells were harvested from femurs and tibiae of 5-week-old, female and male C57BL/6 mice and cultured in complete culture medium (RPMI 1640 supplemented with 10% FBS, 25 mM HEPES, 5 mM 2-ME, antibiotics, and 10 ng/ml mouse GM-CSF and IL-4) for 7 days, and then BMDCs were harvested from floating cells. BMDCs were stimulated by OVA (200 μg/ml) and LPS (100 ng/ml) overnight, with or without 3O-C12 (10 μM). Then, BMDCs were washed and resuspended in PBS. BMDCs (5×10^5) in 20 μl were transferred intranasally (i.n.) to recipients (C57/B6 mice, male) (n = 3 in control group (not treated); n = 10 in OVA + LPS + DMSO-treated BMDC transfer group; n = 11 in OVA + LPS + 3O-C12-treated BMDC transfer group) on days 1 and 12. Each recipient was challenged with 40 μg OVA i.n. on days 13, 14, and 15. One day after the last OVA challenge, mice were sacrificed and the following was assessed: bronchoalveolar lavage fluid (BALF) total cell number and eosinophil number, lung histology, serum immunoglobulins, DC surface marker expression, and $CD4^+$ T cell cytokine production.

Mouse model of asthma

Balb/c mice (6 weeks of age, male) were divided into three groups: control (not treated) (n=4), asthma (n=15), and asthma + 3O-C12 group (n=6). They were intraperitoneally (i.p.) injected on day 1 and day 13 with OVA (50 μg, Sigma) and aluminum hydroxide (2 mg, Sigma). On day 20 and 22, the mice were i.n. challenged with OVA (40 μg) and i.p. administered with or without 3O-C12 (500 μg). On day 25, 27, and 29, the mice were i.n. challenged with OVA (40 μg) and i.n. administered with or without 3O-C12

(500 μg). One day after the last OVA challenge, mice were sacrificed and the following was assessed: bronchoalveolar lavage fluid (BALF) total cell number and eosinophil number, lung histology, serum immunoglobulins, DC surface marker expression, and $CD4^+$ T cell cytokine production.

Multiple cytokine detection

Cytokines were measured by MILLIPLEX® Multiplex Assays using a Luminex 100 platform (Luminex, Austin, TX) and BioManager software (Bio-Rad, Hercules, CA) for analysis. The following multiplexing kits were purchased from Millipore (Billerica, MA): Mouse Cytokine/Chemokine Panel III, containing IL-20, IL-23, IL-27, IL-33, CCL22, and TIMP-1. Kits were used according to the manufacturer's instructions.

Enzyme-linked immunosorbent assay (ELISA)

Cell supernatants were collected 24 h after exposure to 3O-C12. Levels of the cytokines IL-6 and TNF-α (R&D Systems, Minneapolis, USA) were measured by ELISA kits according to the manufacturer's instructions. Mouse serum was collected after the last immunization, levels of total and OVA-specific IgE, IgG1, IgG2a, IgG2c, IgM, IgA, and IgG2b were determined by ELISA. Briefly, OVA was coated in coating buffer (Biolegend). After washing and blocking, diluted serum samples were added, and then primary antibodies against OVA-specific immunoglobulin (Chondrex) were added. After washing, HRP-labeled secondary antibodies (Southern Biotech) were added, TMB Reagent (Biolegend) was used for color development, and optical density was read at 450 nm.

β-galactosidase activity

spDCs and BMDCs from female and male RA reporter mice were incubated with DDAOG (10 μM) in serum-free Hank's buffer for 30 min to 2 h at 37 °C. The cells were washed three times with Hank's buffer before flow cytometry analysis. Upon excitation, DDAO generates a far-red-shifted fluorescent signal that can be detected by flow cytometry.

Western blots

DCs were collected and lysed on ice with RIPA Buffer (CST) containing Protease/Phosphatase Inhibitor Cocktail (CST) and 1 mM PMSF (CST). Cell extracts were loaded onto Biofuraw™ Precast Gel (Tanon), separated by electrophoresis, and transferred onto NC membranes (Millipore). Signals were generated with High-sig ECL Western Blotting Substrate (Tanon). The antibodies were all purchased from CST company: anti-GSK-3β (27C10) rabbit mAb, anti-Phospho-GSK-3β (Ser9) (5B3) rabbit mAb, anti-Phospho-AKT (Ser473) (D9E) XP® rabbit mAb, anti-AKT (pan) (C67E7) rabbit mAb, anti-GAPDH (14C10) rabbit mAb, and NF-κB Pathway Sampler Kit. Band signals were quantified with the ImageJ software and analyzed with the GraphPad Prism software.

DC/T-cell co-culture assays

BMDCs were isolated by EasySep™ Mouse CD11c Positive Selection Kit (stem cell), according to the manufacturer's instructions, loaded with OVA (200 μg/ml), then mixed

with CD62Lhi/CD44low naive CD4+OT-II T-cells (DC/T cell ratio 1:4) isolated with EasySep mouse CD4+T-cell enrichment kit (StemCell), and co-cultured for six days. Differentiation of Th1-Th2 was determined by FACS analysis of intracellular staining of IL4 and IFN- ɣ in activated CD4+OT-II T-cells.

RNA isolation, library construction, and sequencing

Total RNA from DCs was extracted using the TRIzol Reagent (Invitrogen) according to the manufacturer’s protocol. Poly (A)+ RNA was purified from 5 μg of pooled total RNA using oligo(dT) magnetic beads, sheared into short fragments, and primed for cDNA library synthesis using the TruSeq RNA sample preparation kit per Illumina protocol. After quantitation using a TBS-380 mini-fluorometer (PicoGreen), the samples were clustered (TruSeq paired-end cluster kit, v3-cBot-HS; Illumina) and sequenced on the HiSeq2000 platform (100 bp, TruSeq SBS kit v3-HS 200 cycles; Illumina). Annotations for the entire data set were obtained with Tophat and followed by statistical analysis with Cufflink (Cuffdiff module). The read counts were further normalized into FPKM (Fragments Per Kilobases per Million reads). The FPKM values from the libraries were pairwise compared, and the fold changes were calculated by using RSEM software (version 1.2.7), and DEGs were identified by using the DESeq2 or edgeR software package (version 3.8.2) (if $P < 0.05$ and $FDR < 0.05$, the result was considered statistically significant). The data were analyzed on the free online platform of Majorbio I-Sanger Cloud Platform (www.i-sanger.com). The heat maps were generated by HemI (Heatmap Illustrator) software from the collected gene ($P < 0.05$).

Quantitative Real-Time PCR

BMDC cDNA was synthesized from 2 mg of total RNA by the Superscript III reverse transcription system (Invitrogen). qPCR was performed with SYBR green PCR mix and StepOnePlus machine (Applied Biosystems). PCR primers of mouse genes were purchased from OriGene. Gapdh was used as an internal control.

Statistical analysis

Statistical analysis was performed with the SPSS statistical software version 13.0 (SPSS Inc., Chicago). All plot graphs show means with standard deviation (SD). Statistical significance was determined with the two-tailed Student's t-test. A p-value of less than 0.05 was considered statistically significant. *$p < 0.05$, **$p < 0.01$, ***$p < 0.001$, not significant (N.S.). Data that did not exhibit a normal distribution were analyzed using the nonparametric Kruskal-Wallis test with Dunn's post hoc test.
